# Specific Seminal Plasma Fractions Are Responsible for the Modulation of Sperm–PMN Binding in the Donkey

**DOI:** 10.3390/ani11051388

**Published:** 2021-05-13

**Authors:** Jordi Miró, Jaime Catalán, Henar Marín, Iván Yánez-Ortiz, Marc Yeste

**Affiliations:** 1 Equine Reproduction Service, Department of Animal Medicine and Surgery, Faculty of Veterinary Sciences, Autonomous University of Barcelona, ES-08193 Bellaterra (Cerdanyola del Vallès), Spain; dr.jcatalan@gmail.com (J.C.); henarmarin@hotmail.com (H.M.); ivan.yanez22@gmail.com (I.Y.-O.); 2 Biotechnology of Animal and Human Reproduction (TechnoSperm), Institute of Food and Agricultural Technology, University of Girona, ES-17003 Girona, Spain; marc.yeste@udg.edu; 3 Unit of Cell Biology, Department of Biology, Faculty of Sciences, University of Girona, ES-17003 Girona, Spain

**Keywords:** sperm, inflammatory reaction, polymorphonuclear neutrophils (PMN), sperm–PMN binding, donkey

## Abstract

**Simple Summary:**

Previous research demonstrated that seminal plasma, the fluid that together with sperm composes the semen, modulates the interaction between sperm and polymorphonuclear neutrophils (PMN) within the donkey uterus. This study sought to evaluate whether a specific set of proteins or the entire seminal plasma is involved in that modulation. Seminal plasma was fractioned into six portions: <3, 3–10, 10–30, 30–50, 50–100, and >100 kDa. Sperm incubated with two specific fractions, containing 30–50 kDa and 50–100 kDa proteins, exhibited the highest motility and viability after co-incubation with PMN for 4 h, and the highest sperm–PMN interaction at the beginning of the experiment. These results suggest that proteins of 30–50 and 50–100 kDa, rather than the entire seminal plasma, appear to be involved in the inflammatory reaction within the donkey endometrium.

**Abstract:**

While artificial insemination (AI) with frozen-thawed sperm results in low fertility rates in donkeys, the addition of seminal plasma, removed during cryopreservation, partially counteracts that reduction. Related to this, an apparent inflammatory reaction in jennies is induced following AI with frozen-thawed sperm, as a high amount of polymorphonuclear neutrophils (PMN) are observed within the donkey uterus six hours after AI. While PMN appear to select the sperm that ultimately reach the oviduct, two mechanisms, phagocytosis and NETosis, have been purported to be involved in that clearance. Remarkably, sperm interacts with PMN, but the presence of seminal plasma reduces that binding. As seminal plasma is a complex fluid made up of different molecules, including proteins, this study aimed to evaluate how different seminal plasma fractions, separated by molecular weight (<3, 3–10, 10–30, 30–50, 50–100, and >100 kDa), affect sperm–PMN binding. Sperm motility, viability, and sperm–PMN binding were evaluated after 0 h, 1 h, 2 h, 3 h, and 4 h of co-incubation at 38 °C. Two seminal plasma fractions, including 30–50 kDa or 50–100 kDa proteins, showed the highest sperm motility and viability. As viability of sperm not bound to PMN after 3 h of incubation was the highest in the presence of 30–50 and 50–100 kDa proteins, we suggest that both fractions are involved in the control of the jenny’s post-breeding inflammatory response. In conclusion, this study has shown for the first time that specific fractions rather than the entire seminal plasma modulate sperm–PMN binding within the donkey uterus. As several proteins suggested to be involved in the control of post-AI endometritis have a molecular weight between 30 and 100 kDa, further studies aimed at determining the identity of these molecules and evaluating their potential effect in vivo are much warranted.

## 1. Introduction

Jackasses show high spermatogenic efficiency and a relatively short spermatogenesis in comparison with stallions [1]. In general, fresh donkey semen shows good sperm quality characteristics (i.e., reduced sperm abnormalities, high sperm viability and motility, and high proportions of fast, linear sperm motile subpopulations) [2]. Frozen-thawed donkey sperm exhibit good sperm survival and motility [3,4,5], are able to penetrate zona pellucida-free bovine oocytes matured in vitro and form the male pronucleus, and achieve acceptable pregnancy rates when used to inseminate mares. However, their reproductive performance is poor when jennies are inseminated [6,7,8,9].

In mammals, natural breeding or AI induces physiological endometritis. This endometrial inflammatory response is characterized by an apparent infiltration of polymorphonuclear neutrophils (PMN) into the uterus, which is induced by spermatozoa rather than bacteria [10]. The extent of that post-AI endometritis depends on the semen deposition site. In effect, species with vaginal semen deposition, such as ruminants or humans, show a reduced inflammatory response, as thousands of millions of spermatozoa are deposited in the cranial part of the vagina, but the cervix acts as a filter and thus only thousands of sperm cells enter the uterus [11,12]. By contrast, in species with intrauterine deposition, such as rodents, pigs, horses, and donkeys, thousands of millions of spermatozoa are deposited into the uterus and PMN represent the main filtration/clearance mechanism. In the horse, frozen-thawed sperm induces higher post-AI endometritis than fresh semen [10]. In the donkey, the inflammatory endometrial reaction induced by frozen-thawed sperm is even higher than in the horse, as large numbers of PMN and some eosinophils are present in the uterine lumen 6 h after AI [4,13]. Deposited semen is responsible for the uterine inflammatory response inducing strong chemotaxis of PMNs as well as cytokine expression just after insemination [14,15,16,17]. A recent study analyzing sperm–PMN interaction in the donkey showed that while only a reduced percentage of spermatozoa are phagocytosed by PMN, most remain attached on the PMN surface or are found in a surrounding PMN halo [18].

On the other hand, seminal plasma, a complex fluid that contains ions, sugars, lipids, hormones, amino acids, and growth factors [19], is produced by the epididymis and accessory sex glands, including ductus deferens ampullae in the donkey [20]. Composition of seminal plasma differs between species [21], and also between individuals [22]. In effect, apart from the levels of glucose, proteins, lipids, cholesterol, calcium, and phosphorus in seminal plasma being higher in the donkey than in the horse [23], the total protein content of donkey seminal plasma is 4–10 times higher than that of the horse counterpart [23,24]. Furthermore, addition of seminal plasma better maintains post-thaw sperm quality from poor-freezing stallions [25]. Furthermore, while the addition of seminal plasma to frozen-thawed donkey sperm does not appear to improve their characteristics in vitro [5], it increases pregnancy rates following artificial insemination in jennies [26]. Related with this, not only does seminal plasma decrease sperm–PMN binding [13], but it also downregulates COX2 expression in the donkey endometrium [27], thereby supporting a relevant role in the modulation of the endometrial inflammatory reaction upon semen deposition.

While, as aforementioned, the modulating role of seminal plasma on sperm–PMN binding is apparent, it is not clear whether this fluid as a whole or specific fractions, are involved in the control of post-AI endometritis in donkeys. Therefore, this study evaluated the effects of different seminal plasma portions on sperm–PMN binding after fractioning this fluid into separate parts based on molecular weight (3, 3–10, 10–30, 30–50, 50–100, and >100 kDa).

## 2. Materials and Methods

### 2.1. Animals

Five Catalonian jackasses, aged 3–11 years old, and three jennies, aged 5–8 years old, were involved in this study. All animals were in good health and body conditions, and of proven fertility (i.e., semen from males resulted in good fertility rates and females had foaled at least one time). Animals were housed at the Experimental Farm, Faculty of Veterinary Medicine, Autonomous University of Barcelona (Bellaterra, Cerdanyola del Vallès; Spain). Jackasses were allocated to individual paddocks, whereas jennies were maintained in a big paddock (ten individuals per paddock). Animals were fed grain forage, straw, and hay, and water was provided ad libitum. This study was approved by the Ethics Committee, Autonomous University of Barcelona (Code: CEEAH 1424).

### 2.2. Obtaining Seminal Plasma Samples

Three ejaculates per male were collected with an artificial vagina (Hannover model) equipped with an in-line filter (Minitüb Ibérica, S.L.; Reus, Spain). Raw semen was split into 50 mL conical tubes and then centrifuged at 3000× *g* and 4 °C for 10 min (JP Selecta S.A.; Barcelona, Spain). Supernatants were transferred into new 50 mL conical tubes and again centrifuged. Following this, supernatants, presumably containing seminal plasma, were examined under a phase-contrast microscope (Olympus Europe, Hamburg, Germany) to ensure that no sperm was present. This action was repeated as many times as needed (between five and seven, depending on the ejaculate) until samples were sperm-free. The number of centrifugation cycles ranged between five and seven, depending on the ejaculate. Seminal plasma samples were stored in 15 mL tubes at −80 °C.

### 2.3. Fractioning Seminal Plasma Samples

Seminal plasma samples from three ejaculates collected from five different jackasses were thawed on ice, pooled, and fractioned through ultracentrifugation with filters for protein purification and concentration based on molecular weight (Amicon filters, Merck KGaA; Darmstadt, Germany), following manufacturer instructions. Seminal plasma was centrifuged at 4000× *g* and 20 °C for 10 min through a specific filter and a programmable centrifuge (Medifriger BL-SA, JP Selecta, S.A.; Barcelona, Spain) to obtain the <3 kDa fraction. The resulting supernatant was used to obtain the next fraction by another specific filter, and this process was repeated for each fraction. Upon completion of the entire process, six fractions were obtained: <3 kDa, 3–10 kDa, 10–30 kDa, 30–50 kDa, 50–100 kDa, and >100 kDa.

### 2.4. Isolation of PMN

Peripheral blood was collected from three different jennies via jugular venipuncture with 10 mL BD Vacutainer R tubes containing 18.0 mg of ethylenediaminetetraacetic acid (EDTA; BD-Plymouth, UK). Eight tubes from each jenny were collected, and PMN were subsequently isolated following the protocol described by Miró et al. [18]. Briefly, each blood sample was incubated in EDTA tubes for 30 min in a water bath at 37 °C, to stack red blood cells. The leukocyte-rich plasma layer was aspirated, placed in 15 mL tubes, and centrifuged at 402× *g* and 4 °C for 5 min (modified from Loftus et al. [28]). The supernatant was aspirated and discarded; the pellet was resuspended in 2 mL ice-cold PBS, and centrifuged at 402× *g* and 4 °C for 5 min. Thereafter, the supernatant was aspirated and discarded, and the upper white layer of the pellet, which contained neutrophils, was collected and transferred into a 1.5 mL tube. Concentration of PMN/mL was determined with a hematological cytometer (ADVIA 120 Siemens Medical Solutions; Fernwald, Germany), and then adjusted to 100×10^6^ PMN/mL through dilution with ice-cold PBS.

### 2.5. Semen Collection and Preparation

Semen samples were collected with an artificial vagina (Hannover model) equipped with an in-line filter (Minitüb Ibérica S.L.) to obtain a gel-free semen sample. Semen volume was recorded and sperm concentration was evaluated using a Neubauer chamber (Paul Marienfeld GmbH & Co. KG; Lauda-Köngshofen, Germany). Ejaculates were diluted in a skim-milk-based extender [29] and divided into two fractions. One fraction was adjusted to 500 × 10^6^ sperm/mL, whereas the other was centrifuged through a single layer of a silane-coated silica-based colloid (Equicoll, Uppsala, Sweden) in order to eliminate the seminal plasma proteins attached to the sperm surface. Semen was filtered through Equicoll using the protocol for small tubes described by Morrell et al. [30]. Briefly, 1.5 mL of extended semen was carefully pipetted on top of 4 mL of Equicoll in a 15 mL tube. Four tubes under the same conditions were centrifuged at 300× *g* and 20 °C for 20 min. Supernatants and colloids were discarded, and sperm pellets were diluted with the same skim-milk-based extender and adjusted to a final concentration of 500 × 10^6^ sperm/mL.

### 2.6. Co-incubation of Sperm and PMN

Seven treatments, all containing the same PMN and sperm concentrations (100×10^6^ PMN/mL + 100 µl 500×10^6^ sperm/mL; A, B, C, D, E, F, and G), were prepared. Six treatments were added with the obtained seminal plasma fractions (A, B, C, D, E, F); the other one was used as a control and did not contain seminal plasma (G). Detailed description of these seven treatments is as follows: A) 100 µL 100 × 10^6^ PMN/mL + 100 µL 500 × 10^6^ sperm/mL + 100 µL <3 kDa fraction; B) 100 µL 100 × 10^6^ PMN/mL + 100 µL 500 × 10^6^ sperm/mL + 100 µL 3–10 kDa fraction; C) 100 µL 100 × 10^6^ PMN/mL + 100 µL 500 × 10^6^ sperm/mL + 100 µL 10–30 kDa fraction; D) 100 µL 100 × 10^6^ PMN/mL+ 100 µL 500 × 10^6^ sperm/mL + 100 µL 30–50 kDa fraction; E) 100 µL 100 × 10^6^ PMN/mL+ 100 µL 500 × 10^6^ sperm/mL+ 100 µL 50–100 kDa fraction; F) 100 µL 100 × 10^6^ PMN/mL+ 100 µL 500 × 10^6^ sperm/mL+ 100 µL >100 kDa fraction; and G) 100 µL 100 × 10^6^ PMN/mL+ 100 µL 500 × 10^6^ sperm/mL (control). All semen samples were incubated in a water bath at 38 °C, and their viability, motility, and sperm–PMN binding were evaluated at 0, 1, 2, 3, and 4 h. This experiment was repeated 15 times. In each experiment, PMN were obtained from peripheral blood of one jenny (*n* = 3), semen was obtained from one jackass (*n* = 5), and seminal plasma consisted of a pool from the five used jackasses.

### 2.7. Evaluation of Sperm Viability and Morphology

Viability and morphology of the two sperm populations (bound-to-PMN and unbound) were evaluated following eosin-nigrosin staining [31]. Two replicates of 200 spermatozoa each were analyzed under a bright-field, optical microscope (Olympus Europe, Hamburg, Germany) at 1000× magnification. Membrane-intact spermatozoa were considered as viable, whereas morphologically normal spermatozoa were distinguished from those with abnormalities (i.e., tail/head malformations, cytoplasmic droplets, among others). Data were recorded per sperm population (i.e., unbound and bound-to-PMN).

### 2.8. Evaluation of Sperm Motility

Motion characteristics of the unbound sperm population were evaluated through a computer assisted sperm analysis (CASA) system (Integrated Semen Analysis System, ISAS Ver. 1.0.15; Projects and Services R + D, S.L., ProiSer; Valencia, Spain). This system consisted of a negative phase contrast microscope (Olympus BH-2; Olympus Europa, Hamburg, Germany), with a yellow light filter, warm-up plate, and a digital video camera (Basler, Germany) connected to a personal computer containing the ISAS software. Samples (5 µL) were loaded into a pre-warmed (38 °C) Neubauer chamber. Digital images at 200× magnification were recorded and analyzed to remove wrongly detected particles/artefacts. At least 200 spermatozoa were counted and two technical replicates were evaluated.

In each assessment, the following parameters were recorded: curvilinear velocity (VCL), which is the mean path velocity of the sperm head along its actual trajectory (units: µm/s); straight-line velocity (VSL), which is the mean path velocity of the sperm head along a straight line from the first to its least position (units: µm/s); average pathway velocity (VAP), which is the mean velocity of the sperm head along its average trajectory (units: µm/s); linearity coefficient (LIN), which corresponds to VSL/VCL×100 (units: %); straightness coefficient (STR), which results from calculating VSL/VAP×100 (units: %); wobble coefficient (WOB), which is equal to VAP/VCL × 100 (units: %); mean amplitude of lateral head displacement (ALH), which is the mean value of the extreme side-to-side movement of the sperm head in each beat cycle (units: µm); frequency of head displacement (BCF), which is the frequency with which the actual sperm trajectory crosses the average path trajectory (units: Hz); dance, which is equal to VCL/ALH (units: µm^2^/s); absolute mean angular displacement (MADabs), which is the absolute value of the advancing angle of sperm trajectory (units: angular degrees); algebraic mean angular displacement (MADalg), which is the algebraic value of the advancing angle of sperm trajectory, provided that negative values indicate clockwise displacement (units: angular degree).

### 2.9. Determination of Sperm–PMN Binding

Sperm–PMN binding was determined as previously described [32,33]. Ten microliters of each sample was deposited onto a slide, smeared, and subsequently stained with Diff-Quick (QCA, Amposta, Spain). A minimum of 200 spermatozoa per sample were analyzed under a bright-field, optical microscope at 1000× magnification. Two technical replicates were evaluated. The number of spermatozoa attached to PMN was recorded.

### 2.10. Statistical Analyses

Results were analyzed using a statistical package (IBM SPSS 25.0 for Windows; IBM corp., Armonk, NY, USA) and plotted with GraphPad Prism 8.0 for Windows (GraphPad Software Inc.; San Diego, CA, USA). Data were confirmed to be normally distributed (Shapiro–Wilk test) and variances were found to be homogeneous (Levene test). The effects of different seminal plasma fractions (<3 kDa, 3–10 kDa, 10–30 kDa, 30–50 kDa, 50–100 kDa, and >100 kDa) on the viability and motility of unbound and bound-to-PMN sperm populations and sperm–PMN binding (i.e., sperm:PMN ratio, % sperm bound to PMN) were tested through a linear mixed model followed by post hoc Sidak test for pair-wise comparisons. The seminal plasma fraction was the fixed-effects factor, the animal was the random-effects factor, and the incubation time (4 h at 38 °C) was the intra-subjects factor.

Sperm motile subpopulations were set according to the procedure described in Catalán et al. [34]. In brief, individual kinematic parameters recorded with CASA analysis (VSL, VCL, VAP, LIN, STR, WOB, ALH, BCF, and DANCE) were used to run a principal component analysis (PCA). As a result of PCA, which used Varimax procedure and Kaiser normalization, two components, which explained 88.44% of variability, were obtained. Each spermatozoon was assigned regression scores for each of these two PCA components. Based on these scores, spermatozoa were classified into motile subpopulations with a two-step cluster analysis, using the log-likelihood distance and the Schwarz’s Bayesian Criterion. After automatically identifying the number of different subpopulations, which resulted in being four, percentages of spermatozoa belonging to each of these subpopulations for each seminal plasma fraction and incubation time were calculated. These percentages were subsequently used to evaluate the effects of seminal plasma fraction and incubation time with a linear mixed model, as described before.

In all analyses, the level of statistical significance was set at *p* ≤ 0.05. Data are shown as mean ± standard deviation (SD).

## 3. Results

### 3.1. Effect of Seminal Plasma Fractions on Sperm–PMN Binding

**Figure 1:**
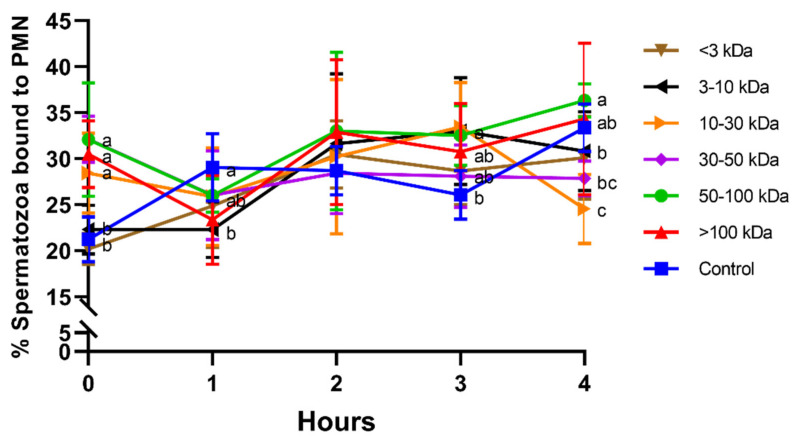
Percentages of spermatozoa bound to PMN for different seminal plasma fractions and throughout incubation at 38 °C for 4 h. Different superscripts (a–c) mean significant differences between seminal plasma fractions within a given time point (*p* ≤ 0.05). Data are shown as mean ± SD for 12 independent experiments.

Treatments including seminal plasma proteins higher than 10 kDa induced a rapid sperm–PMN interaction, with significantly (*p* < 0.05) higher percentage of spermatozoa bound to PMN at 0 h. At 1 h, 2 h, and 3 h, differences between seminal plasma fractions were less apparent. Interestingly, while no significant differences were observed at 2 h, the fraction containing 50–100 kDa proteins showed significantly (*p* < 0.05) higher percentage of spermatozoa bound to PMN than the ones containing proteins smaller than 50 kDa (<3 kDa, 3–10 kDa, 10–30 kDa, and 30–50 kDa) after 4 h of incubation. Moreover, the fraction containing 10–30 kDa proteins showed a significantly (*p* < 0.05) lower percentage of spermatozoa bound to PMN than the control after 4 h of incubation (Figure 1).

**Figure 2:**
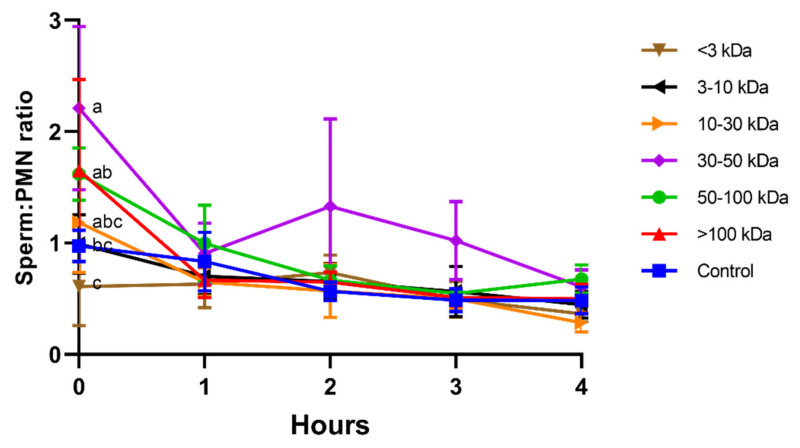
Sperm:PMN ratios (number of spermatozoa attached to one PMN) for different seminal plasma fractions and throughout incubation at 38 °C for 4 h. Different superscripts (a–c) mean significant differences between seminal plasma fractions within a given time point (*p* ≤ 0.05). Data are shown as mean ± SD for 12 independent experiments.

On the other hand, at the beginning of the experiment (0 h), sperm–PMN ratios (i.e., number of spermatozoa attached to one PMN) were significantly (*p* < 0.05) higher in the treatments containing seminal plasma proteins bigger than 10 kDa (Figure 2); in particular, the highest sperm–PMN ratios were observed in the seminal plasma fraction containing 30–50 kDa proteins. However, after 1 h of incubation and until the end of the experiment, no differences between treatments were observed.

### 3.2. Effect of Seminal Plasma Fractions on the Viability of Unbound and Bound-to-PMN Sperm Populations

**Figure 3:**
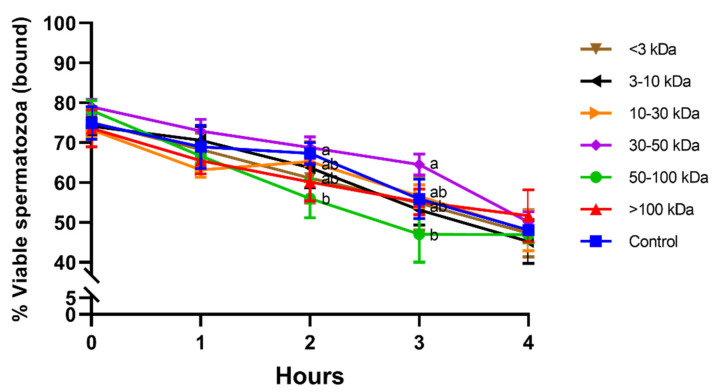
Percentages of viable spermatozoa in the bound-to-PMN for different seminal plasma fractions and throughout incubation at 38 °C for 4 h. Different superscripts (a,b) mean significant differences between seminal plasma fractions within a given time point (*p* ≤ 0.05). Data are shown as mean ± SD for 12 independent experiments.

Figure 3 shows the viability of bound-to-PMN sperm population in the different seminal plasma fractions and throughout the incubation at 38 °C period for 4 h. While no significant differences between treatments were observed at 0 h, 1 h, and 4 h, viability of bound-to-PMN sperm population was significantly (*p* < 0.05) lower in the fraction containing proteins of 50–100 kDa than in the fraction containing proteins of 30–50 kDa after 2 h and 3 h of incubation. In addition, viability of bound-to-PMN sperm was the highest in the fraction containing 30–50 kDa proteins at the same specified times.

**Figure 4:**
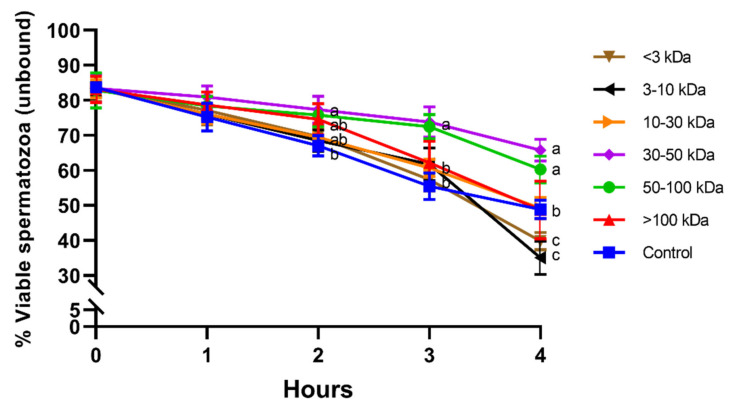
Percentages of viable spermatozoa in the unbound population for different seminal plasma fractions and throughout incubation at 38 °C for 4 h. Different superscripts (a–c) mean significant differences between seminal plasma fractions within a given time point (*p* ≤ 0.05). Data are shown as mean ± SD for 12 independent experiments.

Percentages of viable spermatozoa in the unbound population did not differ between seminal plasma fractions and the control at 0 h and 1 h. Unbound sperm incubated with fractions containing 30–50 kDa or 50–100 kDa proteins showed significantly (*p* < 0.05) higher viability than the control after 2 h, 3 h, and 4 h of incubation at 38 °C. In addition, fractions containing small proteins (<3 kDa and 3–10 kDa) showed a significantly lower percentage of viable spermatozoa in the unbound population than the other fractions and the control at 4 h (Figure 4).

### 3.3. Effect of Seminal Plasma Fractions on the Motility of Unbound and Bound-to-PMN Sperm Populations

In the unbound population, four motile subpopulations were identified. As shown in Table 1, spermatozoa belonging to subpopulation 1 (SP1) showed high velocity (VCL and VAP), very high ALH with high BCF, but low progressiveness (low LIN and STR). Subpopulation 2 (SP2) contained spermatozoa with excellent movement, high velocity (VCL, VSL, and VAP), linear trajectories (high LIN and STR), low ALH, and high BCF. Subpopulation 3 (SP3) included spermatozoa with low velocity but good linear coefficients (LIN and STR), low ALH, and high BCF. Spermatozoa belonging to subpopulation 4 (SP4) exhibited low velocity (VCL and VAP) and low progressiveness.

**Figure 5:**
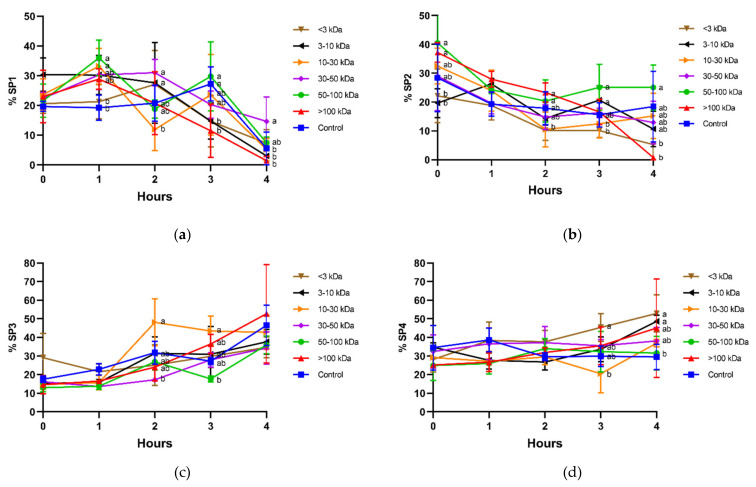
Percentages of motile sperm populations (SP1 (**a**), SP2 (**b**), SP3 (**c**), and SP4 (**d**); unbound sperm) in different seminal plasma fractions and throughout incubation at 38 °C for 4 h. Different superscripts (a,b) mean significant differences between seminal plasma fractions within a given time point (*p* ≤ 0.05). Data are shown as mean ± SD for 12 independent experiments.

In general, incubation decreased sperm motility, as the fastest populations (SP1 and SP2; Figure 5a,b) tended to decrease, and the slowest ones (SP3 and SP4; Figure 5c,d) tended to increase. Interestingly, sperm incubated with seminal plasma fractions containing 30–50 kDa and 50–100 kDa proteins were the ones showing the highest proportions of the fastest populations (SP1 and SP2). This was clear after 1 h of incubation in SP1, as proportions of sperm belonging to that subpopulation were significantly (*p* < 0.05) higher in the presence of 50–100 kDa proteins than in the control, and at 4 h, when the proportions of sperm belonging to SP1 were significantly (*p* < 0.05) higher in the treatment containing 30–50 kDa proteins than in the control.

In the case of SP2, the most linear one, the fraction containing 50–100 kDa proteins was the one that showed the highest proportion of spermatozoa belonging to that subpopulation. In contrast, the proportion of spermatozoa belonging to SP2 in the treatment containing 50–100 kDa proteins was significantly (*p* < 0.05) lower than the control at 4 h.

## 4. Discussion

In mammals, seminal plasma plays an important role for sperm function, both in the male and female reproductive tract [35]. This study provided the first pieces of evidence of the fact that not all seminal plasma proteins exert the same effects upon sperm function and survival and sperm–PMN interaction in donkeys. In effect, we observed that two specific fractions, containing 30–50 kDa and 50–100 kDa proteins, seemed to have positive effects on sperm motility, viability, and sperm–PMN binding, and those containing proteins smaller than 30 kDa had either no effect or a negative impact.

With regard to sperm viability, we conducted the analysis of unbound and bound-to-PMN populations separately. Interestingly, not only did the fraction containing 30–50 kDa proteins show the highest viability values in the bound-to-PMN population, but also in the unbound one. In the case of the fraction containing 50–100 kDa proteins, the effects were less clear, as the viability of bound-to-PMN sperm population was lower than both the control and the fraction containing 30–50 kDa proteins after 2 h and 3 h of incubation at 38 °C. Remarkably, viability of the unbound sperm population was the lowest in fractions containing small proteins (< 3 kDa and 3–10 kDa). Related to this, when they remain in contact with spermatozoa, low molecular proteins (i.e., <10 kDa) are not beneficial for sperm, but rather they reduce motility and damage plasma membrane integrity in several species [35,36,37,38,39]. The effect of these proteins could explain why the addition of seminal plasma semen does not improve frozen-thawed donkey sperm [5].

Sperm motile subpopulations with specific patterns have been previously described in donkeys [2,34,40] and other species, such as common marmosets, gazelles, pigs, dogs, cattle, sheep, horses, and red deer [41,42,43,44,45,46,47,48,49,50,51,52]. Mammals from very different phylogenetic origin show these groups in their ejaculates, suggesting the existence of a relationship between the distribution of these subpopulations and their fertilizing ability [3,45,46,48,50]. In our study, the treatments with seminal plasma fractions including proteins between 30–50 kDa and 50–100 kDa showed the best maintenance of sperm motile subpopulations. Both fractions resulted in high values for the rapid sperm motile subpopulations, SP1 and SP2, without differing with each other. While 30–50 kDa and 50–100 kDa fractions maintained high proportions of SP2, which included fast sperm, they showed reduced proportions of SP3, which contained slow but progressive sperm, and of SP4, which included sperm with non-progressive motility.

While all the aforementioned findings suggest that not all seminal plasma proteins have the same effect, information about these proteins in donkey seminal plasma is scarce, and needs further research. In addition, not only have a reduced number of proteins been described, but their functions also remain mostly unknown. Based on our results and the available literature, one could suggest that some proteins of 30–50 kDa and 50–100 kDa play that beneficial role. Lactate dehydrogenase (LDH) has a molecular weight of 35 kDa, and its levels are high both in horses and donkeys [23,52]. In the horse, the relative amount of LDH, which catalyzes the oxidation of pyruvate to lactate during anaerobic glycolysis, has in seminal plasma been reported to be positively correlated with sperm concentration, motility, and viability, and negatively correlated with morphologically normal spermatozoa [51]. In addition, donkey sperm produce large amounts of lactate, which is positively correlated with motility and velocity, and is thus a good predictor of donkey semen quality [2]. Therefore, LDH could be very important in the control of lactate levels, which would explain why the 30–50 kDa seminal plasma fraction—which would contain LDH—exerts a positive effect on sperm survival and motility.

Moving on to a separate issue, the antioxidant enzyme system involving SOD, CAT, GSR, and GPX has been described in several species [24,53,54,55,56]. A recent study evidenced that the activities of these enzymes are significantly higher in donkey than in horse seminal plasma [24]. This research also found that while activities of SOD, CAT, GSR, and GPX are not correlated with sperm quality in horses, those of SOD and CAT are positively correlated with sperm motility in donkeys [24]. In addition, SOD activity in seminal plasma has been reported to be positively correlated with cryotolerance of donkey spermatozoa [57], and that of GPX has been found to be correlated with age and fertility in Arabian stallions [58]. As the molecular weight of these seminal plasma enzymes ranges between 30 and 100 kDa (CAT: 68 kDa; GSR: 80 kDa; GPX: 88–91 kDa; SOD: 32.5 kDa [59,60,61,62]), one could reasonably suggest that their presence in seminal plasma fractions of 30–50 kDa and 50–100 kDa could explain their positive effects on sperm motility and viability.

Donkey seminal plasma has been demonstrated to be involved in the modulation of sperm–PMN binding [18], and has also been suggested to stimulate PMN to release NETs [63]. Therefore, seminal plasma proteins appear to control the inflammatory response. In this study, we found that, immediately after starting the experiment, not only did seminal plasma fractions containing proteins higher than 10 kDa increase the sperm–PMN interaction, but they also exhibited higher sperm–PMN ratios. We also observed that the seminal plasma fraction containing 50–100 kDa proteins showed a significantly higher percentage of spermatozoa bound to PMN.

Previous studies on sperm–PMN interaction indicate that only a reduced percentage of spermatozoa are phagocytosed by PMN, as most remain attached on the PMN surface or are found within a surrounding halo [18]. Brinkmann et al. [64,65] demonstrated another mechanism of PMN antimicrobial action, through activation and extrusion of their DNA and associated proteins, which allow creating neutrophil extracellular traps (NETs) that block bacteria. Release of NETs in response to sperm–PMN binding has been demonstrated in horses [66] and donkeys [63], among other species. In horses, the DNAse present in seminal plasma has been found to be able to digest the extruded DNA and frees entangled sperm [66]. Since the molecular weight of this DNAse is 33 kDa [66] and we observed that sperm–PMN ratios at the beginning were higher in the seminal plasma fraction containing 30–50 kDa proteins, we suggest that this protein could also modulate sperm–PMN interaction and be involved in NET formation in donkeys. On the other hand, the fraction containing 10–30 kDa proteins showed the lowest percentage of spermatozoa bound to PMN after 4 h of incubation. Related to this, cysteine-rich secretory protein 3 (CRISP3) has been reported to suppress sperm–PMN binding in horses [67]. Although the presence of this protein in donkey seminal plasma has not yet been investigated, it is reasonable to suggest that, given its molecular weight (25 kDa), it could also be present in donkey seminal plasma and could explain why the fraction of 10–30 kDa decreased sperm–PMN binding.

Finally, it should be noted that the current study was conducted using fresh, non-contaminated donkey semen. Kotilainen et al. [10] reported that spermatozoa rather than bacteria induce the influx of PMN into the mare uterus. As we did not observe that PMN bound cells other than spermatozoa, normal semen microbiota does not appear to modulate PMN influx or affect PMN–sperm interaction. However, further studies are needed to elucidate whether alterations in the presence of bacteria in semen interfere with the sperm–PMN interaction and what the impact of the use of antibiotics is.

## 5. Conclusions

In conclusion, this study has reported, for the first time, that specific seminal plasma proteins, notably those of 30–100 kDa, are involved in the modulation of sperm function, including sperm–PMN binding.

## Figures and Tables

**Table 1:** Mean and ranges of different motility descriptors (curvilinear velocity (VCL, µm/s), straight-line velocity (VSL, µm/s), average path velocity (VAP, µm/s), percentage of linearity (LIN, %), percentage of straightness (STR, %), percentage of oscillation (WOB, %), lateral head displacement (ALH, µm), frequency of head displacement (BCF, Hz), DANCE (VCL x ALH, µm^2^/s)), Absolute angular mean displacement (MDA-Abs, Angular degrees) and Algebraic angular mean displacement (MDA-Alg, Angular degrees)) in the identified motile sperm populations.

Motility Descriptors	SP1 (*n* = 6616)		SP2 (*n* = 8484)		SP3 (*n* = 6306)		SP4 (*n* = 9049)	
	Mean ± SD	Range (min, max)	Mean ± SD	Range (min, max)	Mean ± SD	Range (min, max)	Mean ± SD	Range (min, max)
VCL	152.05 ± 29.05	88.95, 304.00	123.42 ± 21.94	68.06, 218.59	54.00 ± 19.74	10.00, 105.92	68.57 ± 21.12	10.14, 132.50
VSL	35.91 ± 20.03	0.00, 135.91	77.62 ± 22.13	27.62, 169.34	31.87 ± 13.45	3.76, 71.49	16.65 ± 9.32	0.00, 46.51
VAP	90.60 ± 22.46	19.47, 230.51	97.40 ± 21.52	51.17, 199.72	38.84 ± 15.30	3.91, 75.03	35.10 ± 13.83	4.20, 81.88
LIN	23.49 ± 11.43	0.00, 63.97	63.25 ± 15.54	28.45, 98.51	59.64 ± 14.75	31.97, 99.21	24.09 ± 10.29	0.00, 44.57
STR	39.83 ± 19.30	0.00, 92.68	79.46 ± 12.37	30.37, 99.72	82.11 ± 10.60	32.47, 100.00	47.76 ± 19.35	0.00, 93.80
WOB	59.90 ± 11.49	11.84, 94.02	79.09 ± 11.69	44.38, 100.00	72.32 ± 12.78	36.74, 100.00	51.03 ± 11.92	9.44, 96.70
ALH	6.08 ± 1.37	2.35, 14.40	3.92 ± 1.21	0.66, 9.70	2.19 ± 0.89	0.23, 5.91	3.24 ± 1.01	0.55, 7.00
BCF	8.24 ± 3.62	0.00, 22.00	8.88 ± 3.10	0.00, 20.00	7.63 ± 3.30	0.00, 20.00	6.40 ± 2.74	0.00, 19.00
DANCE	951.78 ± 389.09	257.76, 4103.72	497.53 ± 215.35	61.09, 1816.66	131.87 ± 85.21	3.37, 479.16	240.97 ± 137.77	5.93, 769.68
MDAabs	111.24 ± 30.92	0.00, 240.19	83.11 ± 44.51	0.00, 266.35	100.79 ± 40.76	0.00, 286.16	122.78 ± 26.64	0.00, 226.72
MDAalg	0.16 ± 9.63	−39.55, 42.46	0.00 ± 9.88	−43.82, 43.71	−0.16 ± 7.77	−41.68, 34.40	−0.25 ± 8.24	−41.01, 43.59

## Data Availability

The data presented in this study are available on request from the corresponding author.
